# Robust Automated Monitoring of Dairy Cow Rumination via Improved YOLOv11 and BoT-SORT in Complex Environments

**DOI:** 10.3390/ani16071109

**Published:** 2026-04-03

**Authors:** Yingjie Zhao, Longjiang Wang, Silei Tang, Qing Zhai, Ruirui Yu, Zongwei Jia

**Affiliations:** College of Information Science and Engineering, Shanxi Agricultural University, Jinzhong 030801, China; z8536495158@163.com (Y.Z.); ww021216www@163.com (L.W.); xtbsh11@163.com (S.T.); 15803301673@163.com (Q.Z.); yuruirui0809@163.com (R.Y.)

**Keywords:** dairy cow rumination, YOLO11, object tracking, attention mechanism, behavior recognition, smart livestock management

## Abstract

Accurate, non-contact monitoring of rumination behavior is essential for assessing dairy cow health and welfare, as well as for optimizing feeding strategies and herd management in modern precision livestock farming. However, practical deployment in commercial barns faces challenges such as occlusions, variable lighting, and dynamic cow movements. To address this, we developed a robust, automated vision-based framework for continuous rumination monitoring. The core of our system integrates an enhanced object detection algorithm with a robust tracking module, specifically improved to capture subtle behavioral features and maintain identity under complex conditions. Evaluated on a comprehensive dataset collected from commercial settings under various lighting and occlusion scenarios, our framework achieved high detection accuracy (mAP of 96.26%) and reliable tracking performance (multi-object tracking accuracy of 99.2%). This demonstrates its suitability for real-time, on-farm deployment. The study provides a practical, end-to-end solution for fine-grained behavioral analysis in complex environments, offering a tool that can enhance welfare assessment and support decision-making in dairy farm management. The methodological approach is also adaptable to other precision livestock monitoring tasks.

## Simple Summary

This study aims to address the critical need for accurate and non-contact monitoring of dairy cows’ rumination behavior, which is crucial for health assessment, welfare improvement, and precision agricultural management. However, due to challenges such as obstruction, changes in light, and dynamic movements, the detection has been hindered. This study is dedicated to developing a reliable automated monitoring solution to overcome these issues, enabling reliable and continuous behavioral analysis to support evidence-based farm management decisions and enhance animal welfare in complex barn environments.

## 1. Introduction

Dairy cows are central to the global dairy industry, and their health and productivity are closely linked to long-term sustainability and economic performance. Rumination is a key physiological activity and a well-recognized indicator of digestive efficiency, energy balance, and early health status [1,2,3]. Accurate and continuous rumination monitoring is therefore essential for optimizing feeding strategies, improving animal welfare, and advancing intelligent livestock management.

Traditional rumination monitoring depends largely on manual observation, which is labor-intensive, subjective, and unsuitable for large-scale or long-term data collection in modern intensive farming systems [4]. Contact-based sensors, such as accelerometers or pressure sensors mounted on ear tags or nosebands, have been used to capture jaw movements or pressure patterns and generally show strong agreement with manual assessments [5,6,7,8]. However, such devices may cause discomfort, skin irritation, or behavioral disturbance, which can ultimately reduce data accuracy and reliability.

In recent years, non-contact computer vision methods have gained increasing attention because they are non-invasive, low-stress, and easy to deploy. These methods typically identify rumination by analyzing motion in the mouth region within video sequences. For example, Benaissa et al. analyzed rumination rhythms using motion features [9], and Wang et al. combined YOLOv5s with DeepSort to track jaw movements for effective rumination monitoring [10]. Despite these advances, real-world farm environments remain challenging. Variable lighting, frequent occlusion, and natural head movements often make the mouth region a small and highly variable target, greatly reducing the robustness of detection models and the stability of tracking algorithms.

To address these limitations, this study introduces an automated analysis framework that integrates an enhanced YOLOv11 detector with the BoT-SORT tracking algorithm to achieve accurate and robust rumination recognition under complex farm conditions. A multi-scenario rumination video dataset was constructed to support model training and performance evaluation. The proposed YOLO11-AT model integrates the Deformable Large Kernel Attention (Deformable-LKA) module and the Vision Transformer with Deformable Attention (DAttention, DAT) to enhance feature extraction and localization for small-scale targets. In addition, the integration of BoT-SORT enables stable cross-frame tracking of mouth movements and supports quantitative analysis of chewing trajectories and rumination frequency. Together, these components provide a reliable non-contact monitoring solution that enhances fine-grained behavior analysis and supports the advancement of smart livestock management.

## 2. Dataset

### 2.1. Video Data Collection and Preprocessing of Dairy Cow Rumination

**Figure 1:**
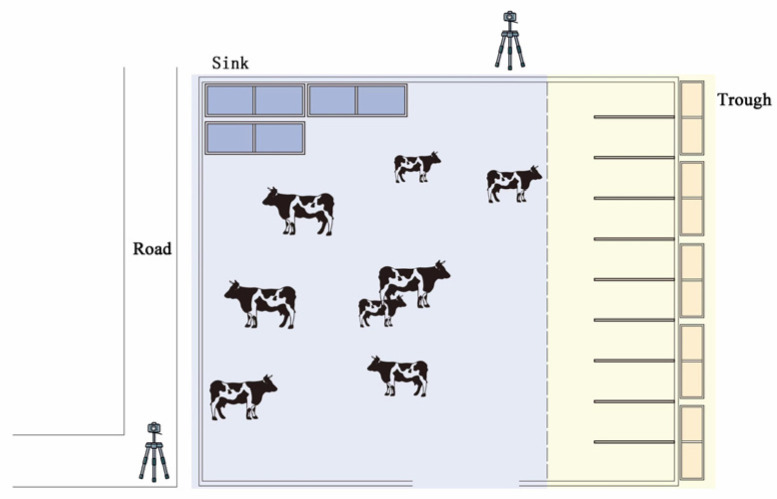
Schematic diagram of the video acquisition platform.

Data collection for this study was carried out from June to July 2022, at the Puyuantai Dairy Farm in Taigu District, Jinzhong City, Shanxi Province. Twenty healthy adult Holstein cows were randomly selected as experimental subjects to ensure adequate sample representativeness. The video acquisition system included a Sony HD camera mounted on a tripod (FDR-AX45, 5044 × 3056 resolution, the video storage format is all AVI, the frame rate is 25.00 fps, Country of origin: Tokyo City, Japan) and a remote monitoring camera with 4G transmission capability. All cameras were placed at fixed locations within the barn to ensure complete coverage of the cows’ primary activity areas, as shown in Figure 1.

**Figure 2:**
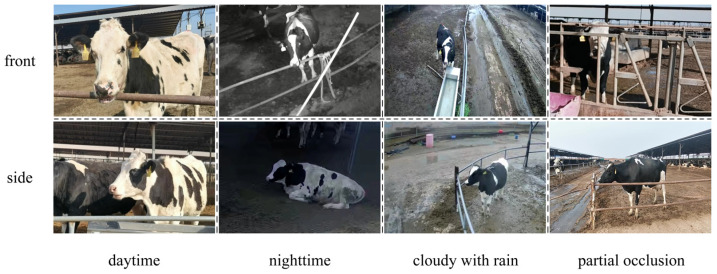
Some data in different scenarios.

To develop a diverse dataset with good generalization, video recordings were captured from two viewpoints, namely front and side, and under four environmental conditions, which included daytime, nighttime, cloudy with rain, and partial occlusion. Representative examples of these conditions are shown in Figure 2. The recorded videos were processed using Free Video to JPG Converter (V5.0.58.324 version), and one frame was extracted every 20 frames, resulting in an initial collection of 3820 images. All images were resized to 608 × 608 pixels to meet the input requirements of the detection model. To enhance robustness to lighting variation, grayscale normalization was performed to standardize image brightness.

**Figure 3:**
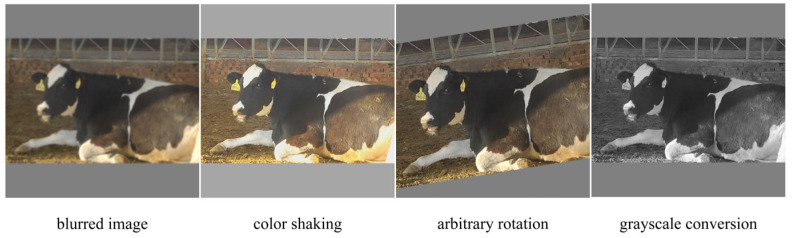
Example image enhancement.

**Figure 4:**
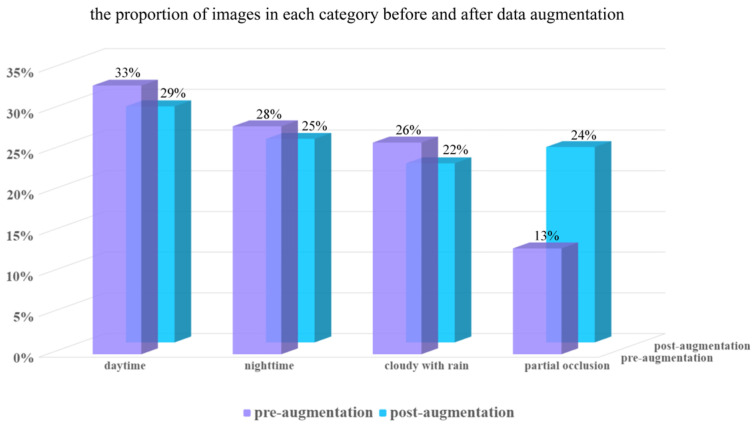
The proportion of images in each category before and after data augmentation.

To address intra-class variation and class imbalance commonly observed in real farm environments, multiple data augmentation methods were applied. These included blurred image, color shaking, arbitrary rotation, and grayscale conversion. Examples of the augmented images are presented in Figure 3. After augmentation, the dataset expanded from 3820 to 6491 images. A comparison of image counts under the different environmental conditions before and after augmentation is provided in Figure 4 (during the daytime, there were 1882 pieces of data; at nighttime, there were 1622 pieces; on cloudy with rain days, there were 1429 pieces; and in partial occlusion, there were 1558 pieces).

### 2.2. Dataset Construction and Annotation

**Figure 5:**
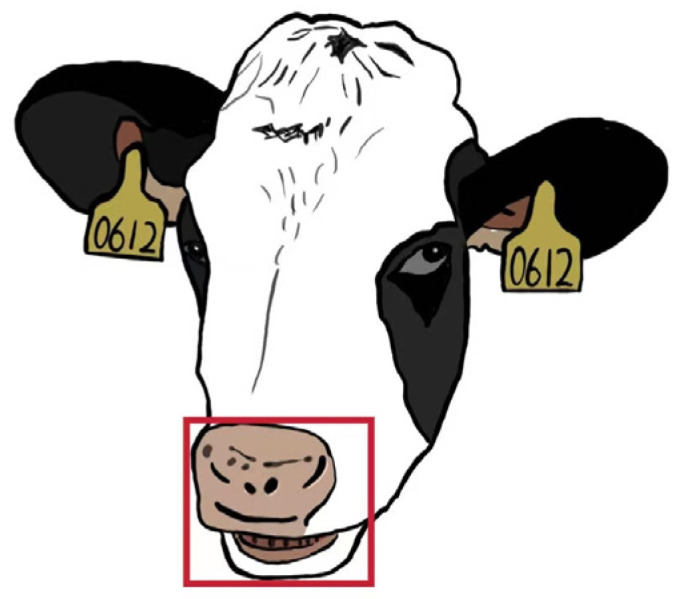
A map marking the area around a cow’s mouth.

The expanded dataset of 6491 images was manually annotated using the LabelImg tool (v1.8.6), and “mouth” was defined as the only target category. A standardized annotation protocol was applied to accurately capture the chewing region. The upper boundary of each bounding box was aligned with the upper edge of the nostrils, the lower boundary extended to the lower edge of the lower lip, and the left and right boundaries were aligned with the outermost corners of the mouth. An example of the annotation format is presented in Figure 5.

After annotation, the dataset was randomly divided into training, validation, and test subsets in an 8:1:1 ratio. The final split contained 5193 training images, 649 validation images, and 649 test images. This division ensured reliable model training and evaluation.

## 3. Improved YOLO11-AT and BoT-SORT Algorithms

### 3.1. Improved YOLO11-AT Model

**Figure 6:**
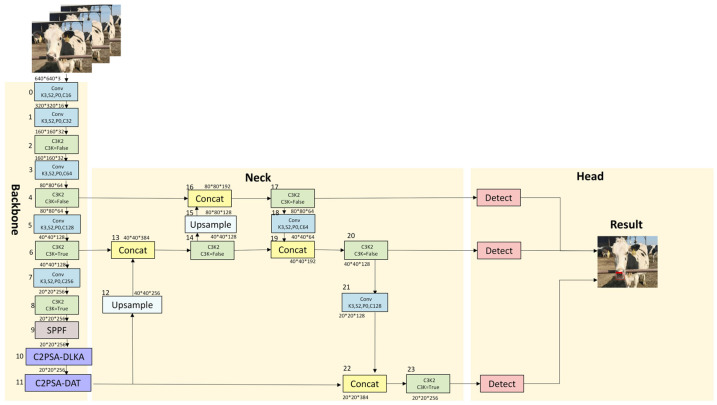
Architecture of the YOLO11-AT network.

To address the detection challenges posed by the small size, variable morphology, and high environmental sensitivity of the cow mouth region, the proposed YOLO11-AT model integrates two key enhancements: the Deformable-LKA module and the DAttention. These improvements are designed to enhance feature extraction and localization accuracy for small-scale targets, forming the core of the YOLO11-AT model presented in this paper [11,12]. The overall architecture of the model is illustrated in Figure 6.

#### 3.1.1. The Integration of the Deformable-LKA Mechanism for Attention

The Deformable-LKA mechanism was integrated into the backbone network, specifically augmenting the feature extraction capabilities following the SPPF module [13,14,15]. The original SPPF module employs multiple parallel max-pooling operations with varying kernel sizes to capture multi-scale contextual information, as defined in Equation (1).

(1) $$F_{\mathrm{SPP}}=\mathrm{Concat}(P_{1\times 1}(F),P_{3\times 3}(F),P_{5\times 5}\left(F)\right)$$

In this equation, F represents the input feature map, and P_k×k_(F) denotes a k × k max pooling operation. Although the module captures multiscale contextual information, the local nature of its convolutional operations restricts its ability to model long-range dependencies.

**Figure 7:**
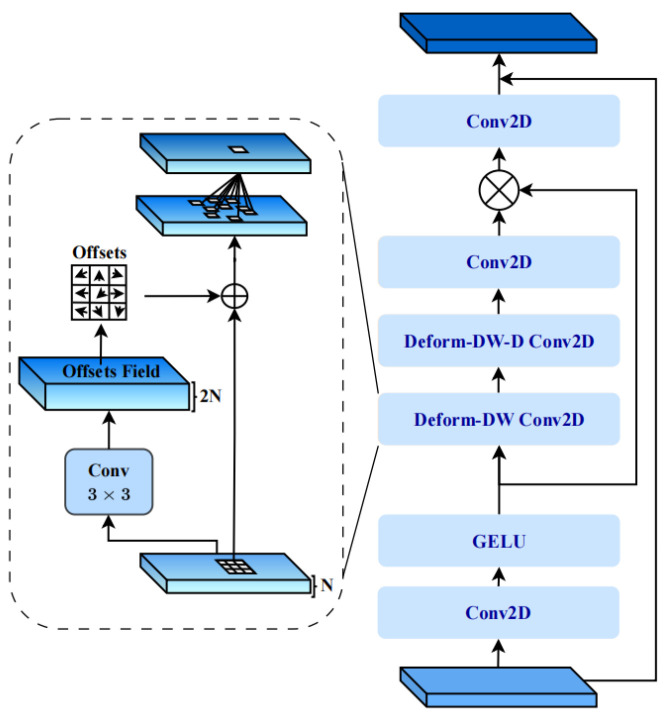
Deformable-LKA module.

To address these limitations, the Deformable-LKA module introduces a deformable large kernel attention mechanism that synergistically combines deformable convolution operations with attention-based feature weighting. As shown in Figure 7, the input feature F is transformed into an adaptively weighted representation through a deformable large kernel attention mechanism that dynamically learns spatial sampling offsets, as defined in Equation (2).

(2) $$F_{\mathrm{DLKA}}=\mathrm{Conv}2D(\mathrm{GELU}(\mathrm{Ddilated}(D(F,\Delta p),\Delta p)))⨂F$$

Through hierarchical deformable attention computation, the module effectively aggregates multi-scale contextual information while maintaining computational efficiency. The inclusion of this module significantly enhances the model’s ability to distinguish and represent target features under complex visual conditions, such as occlusion.

#### 3.1.2. Integration of the DAttention Module

To address the difficulty of detecting and localizing small targets, the Vision Transformer with Deformable Attention (DAttention) module was incorporated at the terminal stage of the backbone network [16,17,18].

Unlike traditional attention mechanisms [19,20,21], which often suffer from high computational complexity in modeling long-range dependencies, and standard Transformer-based attention, which operates on fixed grid locations. The DAttention module introduces a deformable sampling mechanism that dynamically adjusts attention regions based on input content. This design enables efficient capture of both local geometric variations and global contextual relationships while maintaining computational efficiency.

**Figure 8:**
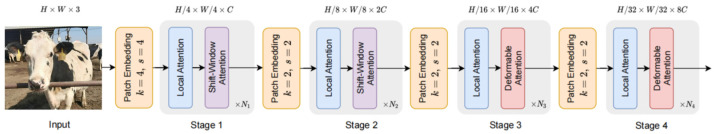
DAttention mechanism.

The structure of the DAttention module is shown in Figure 8. The network takes a color image with dimensions H × W × 3 as input, and first enters the Patch Embedding module of Stage 1. This module performs a large-scale convolution operation on the input image to divide it into non-overlapping local feature blocks and encode them, generating the initial abstract feature map F_1_, as defined in Equation (3).

(3) $$F_{1}={Conv}_{k\times k,stride=4}(I_{in})$$

Here, I_in_ represents the original input image, k × k denotes the size of the convolution kernel, and the step size is set to 4. This step directly reduces the spatial dimensions of the feature map to H/4 × W/4 × C, thereby completing the initial dimensionality reduction in the features and laying the foundation for subsequent multi-scale processing.

After obtaining F_1_, the features will sequentially enter Stages 2 to 4. These three stages adopt the completely identical “feature embedding + dual attention enhancement” structure, which is the core processing unit of the entire network. At the beginning of each stage, the input features will first pass through a Local Embedding module, where they are further extracted with 3 × 3 convolution and batch normalization (BN) to enhance the expression ability of the features, as defined in Equation (4).

(4) $$F_{local}=\mathrm{BN}({Conv}_{3\times 3}(F_{prev}))$$

Here, F_prev_ represents the feature map output in the previous stage.

After local feature encoding, the features are sent to the Dual Attention module. This module consists of two parallel branches, Channel Attention and Spatial Attention, which perform weighted enhancement of the features from the channel and spatial dimensions respectively.

In the Channel Attention branch, first, the spatial dimensions of the feature map are compressed through global average pooling (GAP) to obtain the global statistical information at the channel level [22]. Then, through two layers of fully connected layers and activation functions, the dependency relationships between different channels are modeled, and finally, the channel attention weights are generated, as defined in Equation (5).

(5) $$M_{c}(F_{\mathrm{local}})=\sigma (W_{2}\delta (W_{1}\mathrm{GAP}(F_{\mathrm{local}})))$$

Here, δ represents the ReLU activation function, σ represents the Sigmoid activation function, and W_1_ and W_2_ are the weight parameters of the fully connected layer [23].

This weight will be multiplied with the input features channel by channel, thereby enhancing the feature responses of key channels. In the spatial attention branch, first, GMP and GAP are performed on the feature maps along the channel dimension separately, and the results of these two pooling operations are concatenated along the channel dimension; then, a 7 × 7 convolution layer is used to generate the spatial attention weights, as defined in Equation (6).

(6) $$M_{s}(F_{local})=\sigma ({\mathrm{Conv}}_{7\times 7}([\mathrm{GMP}(F_{local});\;\mathrm{GAP}(F_{local})]))$$

This weight will be multiplied with the input features at each spatial position, thereby enhancing the feature representation of key areas.

Finally, the enhanced features from the two branches of channel attention and spatial attention are added element-wise to obtain the stage output feature map that integrates the dual attention information. As the network progresses from Stage 2 to Stage 4, the spatial size of the feature map will gradually decrease, while the channel dimension will correspondingly expand, achieving multi-scale hierarchical expression of features.

The entire network, through this collaborative effect of multi-stage feature extraction and attention enhancement, ultimately outputs a high-dimensional feature map that can directly serve downstream tasks such as image classification and object detection, significantly improving the model’s ability to capture key information.

### 3.2. BoT-SORT Tracking Algorithm

**Figure 9:**
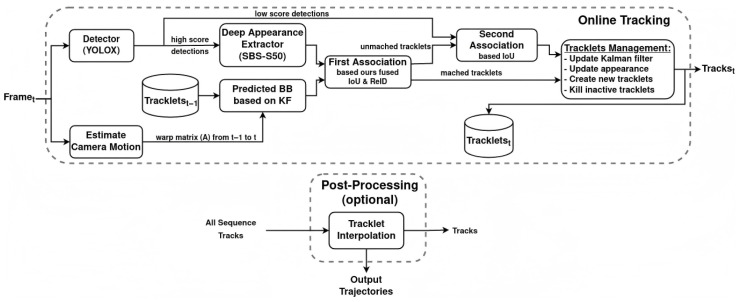
Workflow of the BoT-SORT algorithm.

To ensure stable tracking of mouth movements, the BoT-SORT algorithm was used in this study [24,25,26,27,28]. Built upon SORT [29], BoT-SORT integrates appearance features, camera motion compensation, and an enhanced matching strategy, offering greater robustness than DeepSORT in complex environments [30]. The workflow is illustrated in Figure 9. First, the YOLO11-AT model generates precise mouth bounding boxes for each frame, providing foundational detection inputs. Next, a Kalman Filter predicts target positions in the subsequent frame, while the camera motion compensation module estimates affine transformations to correct coordinate deviations caused by camera movement, eliminating motion induced tracking interference. A two-stage association strategy then links detections to existing tracks: initial IoU-based matching efficiently associates high-confidence detections, and unmatched pairs are re-associated using ReID appearance features to resolve ambiguities from occlusion or deformation. Finally, tracklets are dynamically managed, updating valid associations, creating new tracks for unmatched detections, and removing inactive ones, to maintain consistent target identities across frames. This pipeline yields reliable continuous mouth trajectories, laying a solid quantitative foundation for analyzing chewing behavior.

## 4. Results and Analysis

All experiments were conducted on a workstation equipped with an NVIDIA Tesla K80 GPU with 24 GB of memory. The operating system was Ubuntu 18.04, and the software environment included CUDA 11.7, cuDNN 8.4, Python 3.9.25, and the PyTorch 2.1.0 framework. Model training was performed using the SGD optimizer with an initial learning rate of 0.001, a batch size of 16, and 300 training epochs. An early stopping mechanism was applied to prevent overfitting. The detailed hyperparameter settings are presented in Table 1.

### 4.1. Evaluation Metrics

To comprehensively evaluate model performance, precision (P), recall (R), F1 score, and mean average precision (mAP) were used as evaluation metrics. The corresponding formulations are provided in Equations (7)–(10).

(7) $$Precision=\frac{TP}{TP+FP}$$

(8) $$Recall=\frac{TP}{TP+FN}$$

(9) $$F1=\frac{2Precision\times Recall}{Precision+Recall}$$

(10) $$mAP=\frac{1}{n}\sum_{i=1}^{n}AP_{i}$$

In these formulations, TP, FP, and FN denote the numbers of true positive, false positive, and false negative detections, respectively. All experimental results were obtained on an independent test set to ensure the objectivity of the evaluation.

### 4.2. Model Performance in Ablation Experiments

To verify the effectiveness of each improved module, systematic ablation experiments were conducted on the self-constructed dataset, and the results are presented in Table 2.

Ablation results show that the baseline YOLO11 model achieves an mAP@0.5 of 0.967. When the Deformable-LKA module is introduced independently, its mAP@0.5 drops to 0.955 and the model size increases to 6.7 MB. When the DAttention module is applied independently, mAP@0.5 also decreases to 0.955, with mAP@0.5-0.95 reaching only 0.569. Our proposed YOLO11-AT model achieves balanced performance, with a Precision of 0.933, Recall of 0.884, F1 score of 0.907, mAP@0.5 of 0.962, mAP@0.5-0.95 of 0.578, and a model size of 7.3 MB.

### 4.3. Comparison of Detection Results from Different Methods

To comprehensively assess the advancement of YOLO11-AT, it was compared under identical conditions with several mainstream detection models, including Faster R CNN [31], SSD [32], YOLOv5 [33], and the baseline YOLOv7 and the benchmark YOLO11. All models were trained using the same dataset and hyperparameters to ensure the fairness of the comparison. The experimental results are summarized in Table 3.

As shown in Table 3, both the two-stage detector Faster R-CNN and the single-stage detector SSD perform poorly in the small-scale mouth detection task. Notably, SSD achieves a recall of only 0.671, indicating substantial missed detections, which stems from its reliance on single-scale feature maps that limit its ability to capture the variable appearance of small targets. The YOLO family demonstrates generally stronger performance, with successive iterations delivering continuous improvements: YOLOv7 outperforms YOLOv5 across all metrics, while YOLO11, benefiting from its lightweight design, achieves an mAP@0.5 of 0.967 with a model size of only 5.3 MB. The significant size difference between YOLOv5, YOLOv7, and YOLO11 is mainly attributed to their distinct network architectures. YOLOv7 employs a much deeper and more complex structure with additional advanced modules and richer feature fusion mechanisms, resulting in a larger model size, whereas YOLOv5 adopts a more streamlined design for higher compactness, and YOLO11 achieves a better balance between performance and model complexity through architectural optimization. Our proposed YOLO11-AT has improved the precision to 0.933, which is superior to the 0.918 of the baseline YOLO11. This indicates that the model has higher reliability in predicting positive samples. However, in terms of recall and mAP@0.5, YOLO11-AT is 0.884 and 0.962 respectively, slightly lower than YOLO11’s 0.910 and 0.967. This suggests that the model is basically on par with the baseline model in terms of overall detection coverage and comprehensive positioning accuracy, with a slight trade-off.

Overall, YOLO11-AT achieves a relatively high accuracy while providing higher precision and better stability in complex environments.

**Figure 10:**
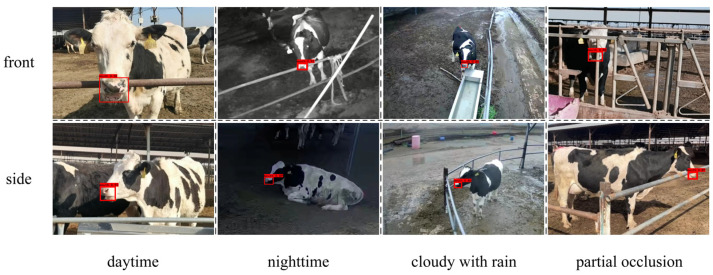
Detection results of mouth regions under different scenarios.

The visualization results under different scenarios, as shown in Figure 10, further demonstrate that the YOLO11-AT model maintains stable detection performance across front and side views, varying lighting conditions, and partially occluded scenes. These results highlight the robustness of the model in practical applications.

### 4.4. Tracking Performance Evaluation

**Figure 11:**
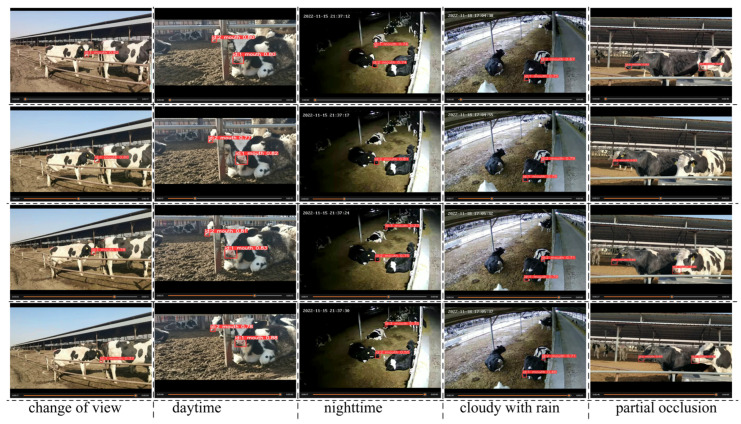
Tracking trajectories of mouth movements during the rumination process.

Using the YOLO11-AT object detection model, the area of the cow’s mouth in the image is obtained. Combined with the BoT-SORT object tracking algorithm, the detected area of the cow’s mouth is tracked for testing, and the ID of the cow’s mouth area is obtained. This paper randomly selected 5 videos to verify the tracking effect of the cow’s mouth area, and the test data included the perspective changes in the cow’s mouth area during rumination (front, side), different environments in the farm (daytime, nighttime, cloudy with rain, partial occlusion), etc., as shown in Table 4. The FPS of the videos was 25 fps. The selected test videos were numbered as 1–5. The results are shown in Figure 11. The visualized tracking trajectory indicates that during continuous chewing, the mouth area shows a consistent periodic movement pattern.

To further quantify tracking performance, comparisons were conducted with mainstream tracking algorithms including DeepSORT, ByteTrack [34], and BoT-SORT. The results are presented in Table 5.

As shown in Table 5, BoT-SORT achieves a MOTA (Multiple Object Tracking Accuracy) of 99.2%, substantially outperforming DeepSORT at 89.8% and ByteTrack at 94.0%. It also records zero identity switches and maintains very low numbers of false positives and false negatives. These results demonstrate that BoT-SORT is highly effective in maintaining identity consistency and trajectory continuity, thereby providing a reliable foundation for the subsequent quantification of rumination behavior.

## 5. Chewing Frequency Estimation Model and Performance Analysis

### 5.1. Determination Model Based on Mouth Movement Trajectories

**Figure 12:**
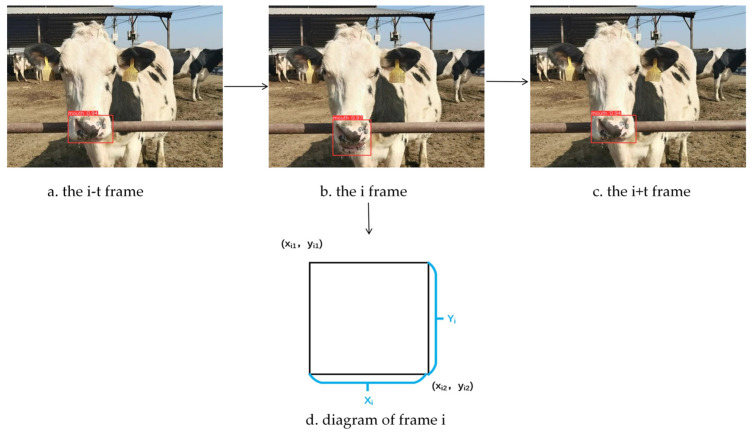
Schematic illustration of the chewing process in dairy cows.

A distinctive feature of dairy cow rumination is the periodic movement of the mouth, characterized by a continuous sequence of closing, opening, and reclosing, as illustrated in Figure 12a–c. The YOLO11-AT model accurately identifies the mouth region and produces detection bounding boxes defined by the coordinates of the upper-left ($x_{i1}$,$y_{i1}$) and lower-right ($x_{i2}$,$y_{i2}$) corners, as shown in Figure 12d. Tracking the variation in these bounding boxes across consecutive frames captures the geometric fluctuations of the mouth induced by chewing movements.

To further quantify the amplitude of mouth movements, the sum of the height and width of the detection bounding box in each frame is calculated to represent the variation pattern of the mouth region throughout the chewing process. The corresponding formulations are provided in Equations (11)–(13).

(11) $$X_{i}=x_{i2}-x_{i1}$$

(12) $$Y_{i}=y_{i2}-y_{i1}$$

(13) $$H_{i}=X_{i}+Y_{i}$$

In these equations, $X_{i}$ denotes the width of the detection bounding box, $Y_{i}$ represents its height, and $H_{i}$ indicates the sum of the height and width.

**Figure 13:**
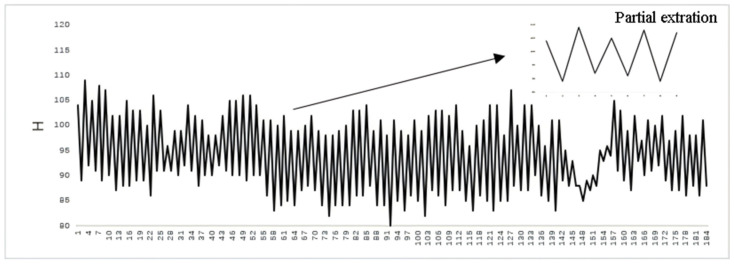
Example chewing curve of a dairy cow.

Figure 13 presents the chewing curve derived from the temporal variation in the mouth-region bounding boxes. The horizontal axis in the figure represents the number of video frames, while the vertical axis represents the sum of the height and width of the tracking bounding box, denoted as H. Each chewing cycle lasts approximately 18 to 25 frames. Since coordinate information is recorded for every frame, noise points inevitably appear. To remove these outliers, the following procedure is applied:

(1) A maximum value within the first 25 frames is identified as the initial peak M_i_:M_i_ = max(1,2,…,25).
(2) Find a minimum value m_i_ in the 12 frames of data starting from M_i_:m_i_ = min(M_i_,M_i+1_,…,M_i+11_).
(3) Find a maximum value M_i+1_ in the 12 frames of data starting from m_i_: M_i+1_ = max(m_i_,m_i+1_,…,m_i+11_).
(4) Repeat steps 2 and 3, and continuously take values and count the number of times and frames the cows ruminate.

**Figure 14:**
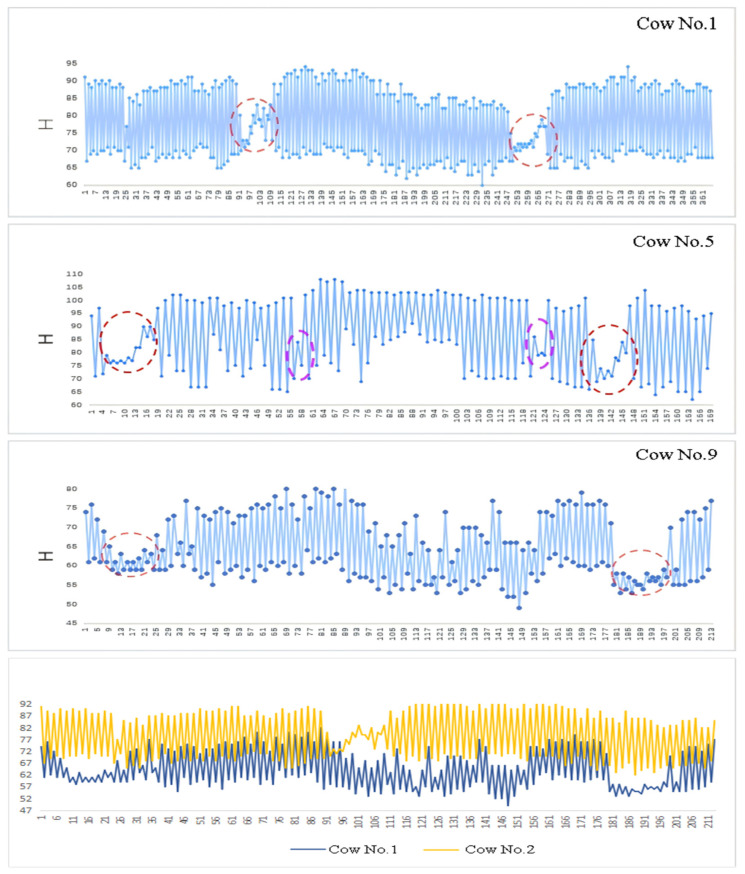
Rumination curves for different targets.

The resulting example rumination curves for single-target and multi-target cows are shown in Figure 14. Figure 14 shows the results of the rumination detection on cows, including the process of a cow chewing a food bolus. The horizontal axis represents the video frame number, and the vertical axis represents the change value H of the tracking box’s height and width. The frames marked by the red box on the curve have a very small change in H, indicating that the height and width of the detection box have changed slightly, which is regarded as the swallowing stage during the rumination period of the cow. The frames marked by the purple box show a decrease in H value within a very short period of time, indicating a change in the rumination angle of the cow. Cow number 5 takes 78 s to swallow a food bolus, while the rumination curves of the double-target cow show that it takes 86 s and 70 s to swallow a food bolus respectively.

### 5.2. Evaluation Metrics for the Rumination Determination Model

(1) Chewing count error rate: defined as the absolute difference between the model-estimated chewing count and the manually recorded chewing count, divided by the manual count. The calculation is given in Equation (14).

(14) $$n_{r}=\frac{\left|m_{r}-p_{r}\right|}{p_{r}}\times 100\%$$

In this equation, n_r_ denotes the chewing count error rate (%), m_r_ represents the chewing count estimated by the model, and p_r_ denotes the chewing count obtained through manual annotation.

(2) Relative error of rumination duration: defined as the absolute difference between the chewing frame count estimated by the model and the manually recorded chewing frame count, divided by the manual frame count. The calculation is provided in Equation (15).

(15) $$n_{t}=\frac{\left|m_{t}-p_{t}\right|}{p_{t}}\times 100\%$$

In this equation, n_t_ denotes the relative error of rumination duration (%), m_t_ represents the chewing frame count estimated by the model, and p_t_ denotes the chewing frame count obtained through manual annotation.

By calculating the chewing counts and frame counts from ten rumination videos recorded during the same time period, the results show an average miscount rate of 3% and an average duration error of 4%. The detailed results are presented in Table 6.

## 6. Discussion

### 6.1. Performance and Innovation Analysis of the Proposed Method

The proposed YOLO11-AT model achieved an mAP@0.5 value of 96.26% and an mAP@0.5-0.95 value of 57.85% when detecting the mouth area of cows in a complex farm environment. Although it has a slightly lower data in terms of recall rate and mAP@0.5 compared to YOLO11, overall, YOLO11-AT can achieve a relatively high accuracy while providing higher precision and better stability in complex environments. This is a more meaningful contribution in the actual farm environment. Specifically, the Deformable-LKA attention module enhances the feature discrimination ability for small targets like the cow’s mouth by dynamically adjusting the receptive field, while the DAttention attention mechanism strengthens the position encoding of key areas through dual-dimensional feature weighting, thereby improving the positioning accuracy of small targets. The synergy of both mechanisms enables the model to exhibit superior performance in small-scale detection tasks. When combined with the BoT-SORT tracking algorithm, this system demonstrates strong performance in target association and identity retention, with a target matching error rate (MOTA) of 99.2%, and no identity switching occurred. This superiority over DeepSORT and ByteTrack stems from the two-stage matching strategy that combines appearance cues with motion modeling, as well as camera motion compensation. In summary, these findings emphasize the importance of combining appearance information specific to a target with precise motion estimation, which is crucial for achieving long-term stable tracking in livestock monitoring applications.

### 6.2. Limitations and Future Work

Although our proposed method has achieved significant improvements in precision, compared to the benchmark YOLO11, its recall rate and mAP@0.5 have decreased. The improvement in precision can be attributed to the introduction of the attention module and the optimized feature extraction structure, which enhance the model’s ability to suppress background interference and reduce false positive detections. However, in the scenario of small target detection, the target features are inherently weak and prone to confusion with the background. Under such stricter conditions, this may unintentionally increase the rate of missed detections, resulting in a slight decrease in recall rate. Since mAP@0.5 simultaneously reflects accuracy and recall rate, the decrease in recall rate further limits the overall improvement of mAP, causing its value to be comparable to the benchmark value.

Furthermore, some limitations must be acknowledged. This system still relies on the quality of the front-end detector, and tracking may be interrupted in cases of severe occlusion. Its performance is also affected by the frame rate and resolution of the video acquisition device, and further evaluation is needed to verify its universality in different farm environments. Additionally, the determination of rumination mainly relies on the geometric changes in the oral area, without fully incorporating temporal context cues, which may lead to misclassification of non-rumination behaviors such as licking or drinking. To address these issues, future research will explore multimodal fusion strategies.

## 7. Conclusions

This study aims to address the challenges in monitoring rumination behavior in complex farm environments. To this end, an automated analysis framework was introduced, which combines the enhanced YOLO11-AT detector with the BoT-SORT tracker. By constructing a multi-scenario dataset and integrating the Deformable-LKA attention mechanism and DAttention attention mechanism, this framework significantly improves the detection and positioning accuracy of small region targets in the mouth. The experimental results show that the proposed YOLO11-AT achieves a relatively high accuracy while providing higher precision and better stability in complex environments, offering a reliable solution for non-invasive rumination activity monitoring. The contributions of this study include developing an optimized small target detection strategy for complex agricultural environments, achieving high-precision quantification of chewing frequency and duration based on vision, and constructing a diverse dataset supporting fine agricultural animal behavior analysis. Future work will focus on exploring multimodal data fusion strategies and optimizing the framework to enable more efficient deployment on edge devices, thereby further enhancing its practicality and universality.

## Figures and Tables

**Table 1:** Hyperparameter settings.

Item	Parameter Setting
optimizer	SGD
epoch	300
batch size	16
early stopping	10
learning rate	0.001
momentum	0.937

**Table 2:** Ablation experiments for mouth region detection.

Improvement Point	P	R	F1	mAP@0.5	mAP@0.5–0.95	Model Size(MB)
YOLO11	0.918	0.910	0.913	0.967	0.587	5.3
YOLO11 + Deformable-LKA	0.928	0.886	0.906	0.955	0.572	6.7
YOLO11 + DAttention	0.892	0.908	0.899	0.955	0.569	5.3
YOLO11-AT	**0.933**	0.884	0.907	0.962	0.578	7.3

Note: Bold values indicate the best results in each column.

**Table 3:** Comparison of mouth region detection results using different methods.

Model	P	R	F1	mAP@0.5	Model Size (MB)
Faster R-CNN	0.894	0.873	0.883	0.908	235
SSD	0.822	0.671	0.738	0.762	244
YOLOv5	0.917	0.870	0.892	0.919	27
YOLOv7	0.921	0.897	0.908	0.942	135
YOLO11	0.918	0.910	0.913	0.967	5.3
YOLO11-AT	0.933	0.884	0.907	0.962	7.3

**Table 4:** Video statistics of cow rumination.

Serial Number	Video Duration/s	Number of Video Frames/f	Number of Dairy Cows	Scene
1	77	1925	1	transition between front view and side view
2	66	1650	2	cow 1: during the daytime, from the front view cow 2: during the daytime, from the side view
3	83	2075	1	at nighttime, from the side view
4	60	1500	2	cow 1: On cloudy with rain, from the side view cow 2: On cloudy with rain, from the front view
5	53	1325	2	partial occlusion

**Table 5:** Comparative results of different tracking algorithms.

Algorithm	MOTA%	FP	FN	IDSw
DeepSORT	89.8	0.0	45.0	6.0
ByteTrack	94.0	28.0	2.0	2.0
BoT-SORT	99.2	0.0	4.0	0.0

**Table 6:** Miscount rate and duration error in tracking chewing counts and frame numbers.

Target Cow	Average Chewing Count		n_r_%	Average Frame Count		n_t_%
	m_r_	p_r_		m_t_	p_t_	
1	21	21	0	637	630	1.11
2	163	163	0	3649	3500	4.26
3	17	17	0	517	512	0.97
4	46	46	0	1170	1175	0.98
5	40	38	5	1489	1488	0.07
6	74	73	1	1914	1800	6.33
7	60	53	13	1377	1175	17.19
8	19	19	0	409	400	2.25
9	32	35	9	640	700	8.57
10	81	79	3	1770	1775	0.28
average	3%			4%		

## Data Availability

The original contributions presented in this study are included in the article. Further inquiries can be directed to the corresponding author.
